# A transient sex-biased transcriptional program shapes early postnatal L2/3 neuron development

**DOI:** 10.1186/s13293-026-00885-x

**Published:** 2026-04-08

**Authors:** Elia Marcos-Grañeda, Fernando Martín-Fernández, Linnea A. Weiss, Juan C. Oliveros, Marta Nieto

**Affiliations:** 1 Department of Molecular and Cellular Biology, Centro Nacional de Biotecnología, Consejo Superior de Investigaciones Científicas (CNB-CSIC), Madrid, 28049 Spain; 2 Present Address: Department of Genetics, Yale School of Medicine, Yale University, New Haven, 06510 CT USA; 3 Bioinformatics for Genomics and Proteomics Unit, Centro Nacional de Biotecnología, Consejo Superior de Investigaciones Científicas (CNB-CSIC), Madrid, 28049 Spain

## Abstract

Most neurodevelopmental disorders (NDDs), including intellectual disability, epilepsy, and autism spectrum disorder (ASD), exhibit marked sex differences in risk, incidence, prognosis, and clinical presentation. Increasing evidence suggests that these disorders often originate from alterations in circuit formation that occur during critical developmental windows preceding the onset of symptoms. However, the extent to which sexual dimorphism emerges during neuronal differentiation remains largely underexplored in the context of NDD research. In the cerebral cortex, layer (L) 2/3 excitatory pyramidal neurons, which integrate information across cortical areas and hemispheres, have been broadly implicated in NDDs. In this study, we investigate the transcriptional expression dynamics of L2/3 neurons across three developmental stages—embryonic day (E) 19, postnatal day (P) 4, and P7—and identify a window of transient, sex-biased gene expression at P4, possibly related to the process of brain masculinization. Furthermore, gene expression analysis following knockout (KO) mutations of *Cux1* and *Cux2* in L2/3 neurons reveals sex-biased dysregulation, with male neurons exhibiting heightened susceptibility and amplified transcriptional disruption, resulting in a molecular fingerprint that is distinct from their WT counterparts. These findings underscore the need to incorporate studies of sex-dependent differentiation trajectories as a biological variable in NDD research and may inform the development of more targeted, sex-specific therapeutic strategies.

**Supplementary Information:**

The online version contains supplementary material available at 10.1186/s13293-026-00885-x.

## Abstract (plain language summary)

Neurodevelopmental disorders like autism, epilepsy, and intellectual disability affect boys and girls differently, but we still don’t fully understand why. One possible reason is that male and female brains develop in slightly different ways, even before symptoms appear. Our study looks at a specific type of brain cell called pyramidal neurons, which receive and process information, and help connect different parts of the brain. We examine these cells in mice at three key stages of early development and discovered that at about four days after birth, male and female neurons show clear differences in the activity of certain genes. We also study the consequences of removing two genes, *Cux1* and *Cux2*, which are important for normal neuronal growth and organization. In their absence, male neurons are far more affected than female ones, showing pronounced changes in gene activity. These findings suggest that sex differences in brain development are important and should be considered when studying conditions like autism or epilepsy. Understanding these differences could lead to better, more personalized treatments for boys and girls in the future.

**Supplementary Information:**

The online version contains supplementary material available at 10.1186/s13293-026-00885-x.

## Highlights

- L2/3 cortical neurons exhibit transient sex-biased expression of autosomal genes at postnatal day 4
- Postnatal day 4 represents a critical point of transient sexual divergence during the differentiation of mouse cortical neurons
- CUX1/2 deficiencies trigger sex-specific dysregulation of sex-independent gene cascades
- CUX1/2 loss-of-function disproportionately affects male neurons during early postnatal development

**Supplementary Information:**

The online version contains supplementary material available at 10.1186/s13293-026-00885-x.

## Introduction

Most neurodevelopmental disorders (NDDs), such as intellectual disability, epilepsy, and autism spectrum disorder (ASD), exhibit significant sex differences in their risk, incidence, prognosis, and clinical presentation [78, 82, 101]. These disparities are not limited to sex chromosome-linked disorders (e.g., Rett syndrome, Fragile X) but are also evident in conditions associated with autosomal genetic variants [39, 80, 91]. Sex-dependent gene regulation, differential interactions with sex hormones, and environmental exposures are all recognized as factors potentially contributing to sex-bias [41, 53]. However, the field lacks mechanistic studies offering an integrative framework for understanding the influence of sex on brain development as well as on NDDs. This gap is particularly critical in the context of autosomal-linked NDDs, where sex differences are less intuitively explained.

The complex functions of the brain require the coordinated assembly of diverse neuronal types and circuits during development, as well as a tight spatiotemporal regulation of gene expression [38]. A single genetic mutation can destabilize the entire system, particularly if occurring early in development. Like a shock-wave or domino effect, alterations in a gene regulatory network can be amplified over time, often obscuring the root causes of the pathological phenotypes observed in NDDs [40].

It is not uncommon that disruptions in distinct genetic or cellular components converge to alter the same process and result in similar malfunctions, and conversely, that various mutations within the same gene can give rise to distinct phenotypes [22, 30, 68]. Compounding this already complex scenario, each sex introduces unique developmental differences that can differentially shape the cascading effects of gene mutations. Supporting this scenario, growing evidence indicates that many NDDs emerge from dysregulations during discrete developmental windows, commonly referred to as critical periods, when the brain is particularly sensitive to biological and environmental factors that can exert lasting effects on structure and function [55, 62]. It is therefore critically important to understand how genetic mutations might derail developmental gene expression in a sex-divergent manner, as well as the precise identification of such windows of sexual divergence.

Mutations in *CUX1* and *CUX2* genes are associated with epilepsy, ASD, intellectual disability, and global developmental delay (GDD) [1, 2, 12, 24, 48, 67, 69]. *CUX1* and *CUX2* encode for proteins that are members of the CUT class of transcription factors (TFs), a highly conserved family of homeobox genes in metazoans. The CUX1 and CUX2 proteins both contain three CUT repeats and a homeodomain [6] and function as transcriptional activators or repressors, depending on the promoter sequences and cellular context [76, 89, 93].

In mammals, *CUX/Cux* genes are expressed throughout the central and peripheral nervous systems (CNS and PNS) during development and into adulthood. Within the CNS, they are present in distinct progenitor and neuronal subpopulations of the dorsal telencephalon, the hypothalamus, and the cerebellum, among other regions [8, 23, 61, 73, 85, 87, 96]. In the cerebral cortex—where their roles have been most extensively characterized—their expression is conserved in pyramidal neurons of the upper layers of the neocortex in both mice and humans [61, 71, 81]. In the mouse hippocampus, *Cux2* marks a subpopulation of perinatal neurogenic progenitors but is not detected in neurons; transcriptomic studies indicate that *CUX2* is also expressed in the human hippocampus [13, 97]. In contrast, CUX1 protein is robustly expressed in the developing human dentate gyrus, whereas comparable studies in mice report little to no *CUX1* RNA or protein in developing or adult hippocampal regions [13, 61].

CUX/Cux homologues contribute to subtype-specific features of neurons in the neocortex, a key structure for high-order functions often affected in NDDs [17, 18, 49, 76]. The neocortex is organized in six layers, with neurons in each layer (L) sharing similar morphology, molecular identity, and connectivity patterns [28]. As mentioned above, the expression of *CUX1/Cux1* and *CUX2/Cux2* is a genetic signature of the excitatory pyramidal neurons that populate the most superficial layers of the brain. Among them, L2/3 neurons are particularly notable for their role in integrating information within different cortical areas and across the hemispheres. They are characterized by complex dendritic arbors and dense dendritic spines, and their long-range axons constitute a major component of the corpus callosum. Studies in the mouse have shown that proper differentiation of L2/3 neurons critically depends on the expression of *Cux1* and *Cux2 *[17, 20, 26].

In mice, *Cux1* and *Cux2* expression begins early in neuronal differentiation. CUX1 and CUX2 proteins are detected in migrating L2/3 neurons shortly after their birth at embryonic stages; their expression is thereafter maintained throughout the neurons’ postmitotic life [61]. In *Cux1* and *Cux2* single mutants, L2/3 neurons laminate normally, but dendritogenesis and synaptogenesis are defective [17]. Specifically, *Cux1* loss of function predominantly affects basal dendrites while *Cux2* mutations alter the apical compartment [18]. In double *Cux1;Cux2* mutants, the dendritic defects are additive and significantly exacerbated [17]. For other aspects of neural development, the two proteins show specific non-redundant actions. For example, CUX1 regulates the development of L2/3 callosal axons [76], whereas CUX2 controls the proliferation of L2/3 and L4 progenitors [16]. Despite their well-established roles in cortical development, little is known about their transcriptional targets or the dysregulation of gene expression caused by their loss in neurons. Consistent with a critical role in brain function, loss of these genes leads to several neurological phenotypes. In humans, patients carrying *CUX1* mutations in heterozygosis present with heterogeneous symptoms, including delayed speech and motor development, seizures, mild cerebellar symptoms, intellectual disability, and ASD [1, 24, 48, 67, 69]. Heterozygous *CUX2* mutations have likewise been identified in patients with rare epileptic encephalopathies, bipolar disorder, and intellectual disability [2, 12]. In mice, *Cux1* knock-out (KO) animals die shortly after birth, demonstrating the lethality of homozygous mutations [52]. Coinciding with human symptoms, heterozygous animals were shown to be more susceptible to seizures in a recent study [67]. In this study, no sex differences were found in mice or humans. On the other hand, defects in working memory have been reported in *Cux2* KO male mice, although the study did not examine females [17]. Overall, the lack of sex-specific analysis in both mouse and human studies, and particularly, the limited number of identified patients with pathogenic CUX mutations together prevented evaluation of potential sex differences in CUX functions, incidence, and clinical phenotypes. It therefore remains unclear whether CUX/*Cux* mutations interact with sex genotype.

In recent years, high-throughput approaches such as RNA-sequencing (RNA-seq) and microarray-based technology, have enabled an in-depth characterization of sex-biased gene expression. Most of these gene expression studies in the brain have focused on the adult, often analyzing gene expression in bulk RNA obtained from brain samples that included a mixture of cellular populations [63, 75, 86, 98]. This approach may mask sex-dependent differences in specific neuronal subpopulations or circuits. Herein, we investigated changes in gene expression specifically in L2/3 neurons. We isolated these cells from male and female mice of wild-type (WT) and *Cux1* and *Cux2* mutant genotypes across the perinatal window delimited by embryonic day (E) 19 and postnatal day (P) 7.

In the present study, we defined the sex-biased gene expression profile of WT L2/3 neurons across perinatal development. From very similar profiles at E19, sex-biased differences peaked at P4 before diminishing at P7. The data thus revealed two distinct sex-dependent differentiation trajectories that converge on a common molecular L2/3 identity by P7. This highlights the first postnatal week as a critical developmental window during which genetic or environmental insults may affect males and females differently. Accordingly, we found a significantly greater impact of *Cux1* and *Cux2* mutations on the transcriptome of male-derived relative to female-derived L2/3 neurons during this critical period. Notably, the sex-biased consequences of *Cux* mutations involved molecular cascades that are normally sex-independent, thus illustrating a sex-dependent derailment of L2/3 neuronal differentiation triggered by a somatic gene mutation. Together, our findings uncover previously unrecognized sex-dependent features of gene expression during perinatal cortical development and provide important insights into how autosomal mutations may affect sexual development to influence NDD risk and phenotype.

## Materials and methods

### Mice

C57BL6J^RccHsd^ mice (Envigo Laboratories, formerly Harlan) were used as wild-type animals. *Cutl1*^tm2Ejn^ (*Cux1*^*∆C*^) mouse line was obtained from A.J. van Wijnen (University of Massachusetts Medical School, Worcester, MA) [52]. *Cux2*^tm1.1Nieto^ (*Cux2*^−^) mice were previously generated and described as shown in [16]. The generation of a *Cux1* flox allele (*Cux1*^*f*^), and subsequently *Cux1*^*f/f*^ and *Cux1*^*f/∆23*^ mice, is detailed in Supplementary Methods. Transgenic mice were maintained in heterozygosis in the C57BL6J^RccHsd^ background. The day of appearance of a vaginal plug was defined as E0.5, and the day of birth, postnatal day 0 (P0). Animals were housed and maintained following the European Union guidelines (directive 2010/63/EU). Procedures were approved by the Ethical Committee of the Spanish National Research Council and the Ethical Animal Research Committee of Madrid following the National and European directives (PROEX: 215/19, 395/15, 124/17, and 058.1/22).

### In utero electroporation

Surgery was performed as previously described [5]. Timed pregnant mice were anesthetized with 3/98% isoflurane/oxygen and maintained at 1.5–2% isoflurane during the procedure. pCAG-GFP (AddGene, plasmid #11150) or a mixture of pCALNL-GFP (Addgene, plasmid #13770) and pCAG-iCre (Addgene, plasmid #89573) were injected at 1 µg/µl with Fast Green (0.1%, #F7252) into the lumen of the embryos´ ventricle system using pulled glass micropipettes. Five voltage pulses (50 ms, 38 V, spaced 950 ms) were applied with platinum tweezer-type electrodes (Sonidel Limited, #CUY650P5) oriented to the target somatosensory (SS) cortex. To collect embryonic samples, a c-section was performed at E19. For postnatal samples, P2 GFP^+^ pups were selected and developed normally until the adequate stage.

### Fluorescence-activated cell sorting and RNA extraction

Successfully electroporated brains were extracted at the specified developmental stages. The fluorescent cortical area was isolated using a microscope attached to a fluorescent lamp and maintained in cold PBS. A tail sample was collected for genotyping and sexing [56] (see Supplementary Methods). Dissected tissue was incubated for 15 min at 37ºC with 250 μl of a D-PBS/liberase solution (70/30%, Roche, #05401119001) and 300 units of DNaseI (Invitrogen, #18047019). Mechanical dissociation by pipetting was also performed. Then, enzymatic digestion was stopped by adding an equal volume of cold D-PBS/0.5% FBS (Merck, #F2442) followed by centrifugation (250 x g, 7 min, 4 °C). Pellets were gently resuspended in 400 μl of cold D-PBS/0.5% FBS with fire-polished glass pipettes. After another centrifugation, pellets were resuspended in 1.5 ml of cold PBS, and 450 μl of Debris Removal Solution (Miltenyi Biotec, #130–109–398) were added. Cell debris was removed following the manufacturer´s protocol. Finally, cells were resuspended in 200 μl of sort buffer (90% D-PBS, 0.5% FBS, 10% trehalose (Sigma, #T9531) and 0.5% DAPI (Merck, #D9542). FACS was performed in a BD FACS Aria II SORP Cell Sorter (BSL-2) at the CNIC (Madrid, Spain). Cells were collected in kit lysis buffer and snap-frozen to store them until used. RNeasy® Plus Micro Kit (Qiagen, #74034) was employed for RNA extraction. Final concentration and quality were determined by microfluidics-based automated electrophoresis using a Bioanalyzer (2100 Bioanalyzer System).

### RNA-sequencing and data processing

RNA libraries were generated in the Genomics Unit of the CNIC (Madrid, Spain). 300 pg of total RNA (RNA integrity number > 7.5) were used to generate barcoded RNA libraries using the NEBNext Single Cell/Low Input RNA Library Prep Kit for Illumina (New England Biolabs, #E6420) according to the manufacturer’s instructions. Low Input RNA sequencing was performed using Illumina NextSeq 2000 (Illumina), and 60 nucleotides single-end reads were obtained. Different replicates per group and developmental stage were sequenced (WT: n = 4 at E19 per condition, n = 3 at P4, n ≥ 2 at P7; *Cux1*^*∆C/∆C*^: n = 4 at E19 per condition; *Cux1*^*∆23/∆23*^: n = 2 at P4; *Cux2*^−/−^: n = 4 at E19 per condition, n = 3 at P4). Single-end reads in FastQ format were quality-checked with FastQC (bcl2fastq 2.20 Software, Illumina) [27]. Data processing was performed using SeqNJoy (https://bioinfogp.cnb.csic.es/tools/seqnjoy/, manuscript in preparation). Unprocessed reads were aligned to the mouse genome (GRCm39, primary assembly) using hisat2 function of Bioconductor package Rhisat2 [42] (min-intronlen 20, max-intronlen 100,000): female samples were aligned to the Y-masked reference genome, and males were aligned to the YPAR-masked reference genome [66]. Duplicated reads were removed using removeDupReads function of Bioconductor package Rsubread [47] (threshold 50). IGV browser [74] was used to visualize alignment results. Mouse genes were quantified using featureCounts function of Bioconductor package Rsubread [47] (primaryOnly TRUE) with Mus_musculus.GRCm39.108.gtf file as genome annotation. Normalized counts were obtained using DESeq function of bioconductor package DESeq2 [50]. Principal component analysis (PCA) and Pearson correlation analysis were then conducted with variance-stabilized read count data using plotPCA function of bioconductor package DESeq2 [50] and pheatmap function of pheatmap package [43] respectively.

### Gene expression analysis

Differential expression analysis was performed for each comparison between conditions (*Cux* and sex genotypes) using DESeq function of bioconductor package DESeq2 [50] (independentFiltering TRUE). Results, including FDR values [4], were stored as Excel files and visualized using FIESTA [65]. log_2_Fold Change (log_2_FC) was used to assess changes in gene expression among conditions. Genes with a specific log_2_FC (|log_2_FC|> 0.5 for E19 and |log_2_FC|> 1 for P4 and P7 analysis) and FDR < 0.05 were considered differentially expressed. Volcano plots showing differentially expressed genes (DEGs) were generated using ggplot function of ggplot2 package [94]. Genes with positive log_2_FC values were considered up-regulated (URGs) while those with negative values were considered down-regulated (DRGs). Venny 2.1 software [90] was used to compare DEGs between conditions to identify common targets.

### Enrichment analysis

Overrepresentation analysis (ORA) of Gene Ontology (GO) terms for the biological process was performed using enrichGO function of R package clusterProfiler [95, 99] (pAdjustMethod = "BH") with a genome-wide annotation package for mouse [9]. DEGs, URGs, DRGs, and common DEGs were used as gene sets. GO categories with a p-value < 0.05 and a minimum count of three genes were considered significant. ggplot function of ggplot2 package [94] was used for data visualization.

## Results

### Sexual divergence in L2/3 neurons gene expression profiles during early postnatal development

Functional studies and clinical reports underscore the importance of *Cux* genes in the development of cortical functions [16–18, 67, 69, 76]. However, little is known about their transcriptional targets in the neocortex. To gain insight into the gene networks regulated by CUX1 and CUX2 in L2/3 neurons, we performed bulk RNAseq analysis of this subpopulation isolated from the somatosensory (SS) cortex of WT and *Cux1* and *Cux2* mutant mice. Homozygous *Cux2* ^−/−^ knock-out (KO) mice are viable, but *Cux1* germline KO mutants (*Cux1*^*∆C/∆C*^) die perinatally [16, 52]. To bypass this lethality, we produced a novel *Cux1* flox allele (hereafter, *Cux1*^*f*^) using CRISPR/Cas9 recombination to introduce two loxP sequences flanking exon 23. This exon encodes part of the homeodomain and the nuclear localization signal of CUX1, and its elimination produces a truncated protein that is rapidly degraded [52]. We confirmed that this approach eliminates expression of the full-length isoform of the CUX1 protein (CUX1 p200) by Western blot (see Supplementary Methods and Fig. S1). We then crossed *Cux1*^*f/f*^ mice with *Sox2*-Cre animals to generate a *Cux1*^*∆23*^ allele in the germline and thereafter, by intercrossing, a double heterozygous flox/KO mouse line (*Cux1*^*f/∆23*^). These double heterozygous mice show no obvious defects or phenotypes, and breed and grow normal. The latter genetic configuration ensures that upon CRE recombinase expression, only a single recombination event is required to generate CUX1-deficient neurons. Consequently, the approach enables efficient gene inactivation upon the expression of CRE.

For RNA analysis, we targeted expression of GFP specifically in L2/3 neurons by in utero electroporation (IUE) of their progenitors in the embryonic (E) 15.5 brain. In WT, *Cux1 *^*ΔC/ΔC*^, and *Cux2* ^−/−^mice, we only electroporated the plasmid encoding GFP. In *Cux1 *^*f/Δ23*^ mice, we added to the DNA mix another plasmid for the expression of CRE, thus selectively inducing the CRE-mediated recombination of the *Cux1* flox allele in the targeted cells to produce KO (*Cux1 *^*Δ23/Δ23*^) L2/3 neurons in viable heterozygous animals. Immunolabeling performed in histological brain sections confirmed the efficient CRE recombination of the *Cux1*^*f*^ allele and the elimination of the CUX1 protein specifically in the GFP^+^ L2/3 neurons of the electroporated animals (Fig. S1). L2/3 neurons obtained from *Cux1 *^*ΔC/ΔC*^ embryos and the postnatal *Cux1 *^*Δ23/Δ23*^ mutant neurons generated by IUE are hereafter referred to as *Cux1* KO L2/3 neurons. Likewise, L2/3 neurons from *Cux2* ^−/−^ mice are referred to as *Cux2* KO L2/3 neurons.

**Fig. 1:**
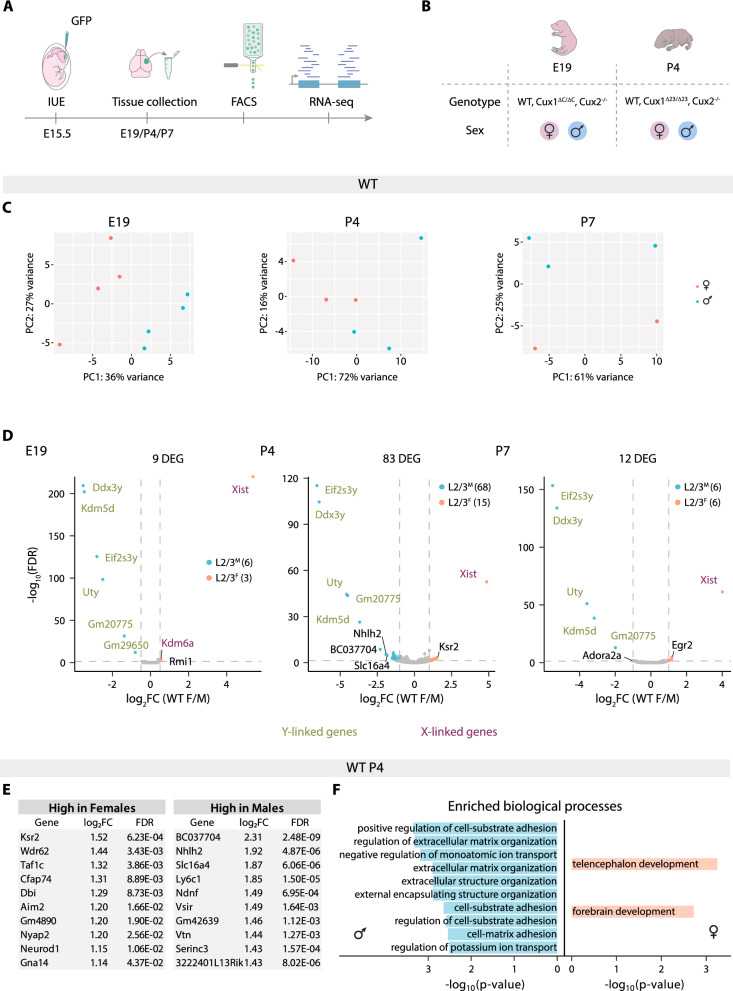
Sexual dimorphism in L2/3 neuronal gene expression peaks at P4. **A)** Schematic representation of the experimental workflow. Mice were subjected to IUE at E15.5 with plasmid to produce GFP. Fluorescent tissue was dissected and processed at different developmental stages (E19, P4 and P7). GFP^+^ cells were then isolated by fluorescence-activated cell sorting (FACS) and total RNA was extracted and submitted for sequencing in two separate rounds (first E19, and second, P4 and P7). **B)** Table summarizing the collected samples for RNA-seq. Female and male neurons of WT genotype were used at all stages. *Cux2* KO neurons were used at E19 and P4. *Cux1*^*ΔC/ΔC*^ neurons were used at E19 and *Cux1*^*Δ23/Δ23*^ neurons at P4. **C)** Principal component analysis of E19 (left), P4 (middle) and P7 (right) of WT samples. Indicated percentages on each axis represent the proportion of variation explained by the principal components. **D)** Volcano plots showing differentially expressed genes (DEGs) in WT L2/3^F^ neurons compared to L2/3^M^ at the indicated developmental stages. The effect size and direction for each gene is depicted in the x-axis as the log_2_Fold Change (log_2_FC). False Discovery Rate (FDR) values are represented on the -log_10_ scale in the y-axis. Orange and light blue dots represent up-regulated and down-regulated genes (URGs and DRGs), respectively, based on the significance threshold. Green and purple names represent Y- and X-linked genes, respectively. Dashed lines mark the threshold in each plot: A, |log_2_FC|> 0.5, FDR < 0.05; B,C, |log_2_FC|> 1, FDR < 0.05. **E)** Tables showing the log_2_FC and FDR values of the top 10 autosomal DEGs in WT L2/3^F^ (left) and L2/3^M^ (right) neurons at P4. **F)** Representation of up to 10 most significant gene ontology biological processes obtained from the DEGs enrichment analysis of WT L2/3^M^ (left) and L2/3^F^ (right) neurons at P4. Color code is maintained from the volcano plots

After IUE, we performed fluorescence-activated cell sorting (FACS) to isolate L2/3 neurons from SS cortices and sequenced RNA to compare their transcriptomic profiles at key developmental stages: (i) E19, an early stage of neuronal differentiation; (ii) P4, the start of neurite and axonal development; and (iii) P7, a stage preceding synaptic and axonal maturation (Fig. 1A). To evaluate sex differences in neuronal differentiation, we obtained female and male samples (Fig. 1B). Hereafter, L2/3 neurons from female and male mice are referred to as L2/3^F^ and L2/3^M^ neurons, respectively.

Unexpectedly, we observed a significantly higher rate of neuronal death among *Cux1* KO L2/3 neurons compared to WTs during tissue dissociation. We hypothesize that this cell death results from a heightened vulnerability of CUX1-deficient neurons to mechanical shearing. The underlying mechanisms contributing to this sensitivity will be investigated in a separate study. This enhanced fragility was more pronounced with maturation and prevented the collection of any RNA samples from P7 *Cux1* KO L2/3 neurons, but we were able to collect samples from P4 *Cux1* KO males. 

We first compared the gene expression profiles of L2/3 and L2/3 neurons from WT animals across developmental stages and sex. A principal component analysis (PCA) revealed that, for each stage, samples cluster according to sex (Fig. 1C). This indicates sex-dependent L2/3 differentiation trajectories in a WT genetic background. Notably, the differences in gene expression (i.e., number of differentially expressed genes (DEGs)) in females and males varied across postnatal differentiation. At E19, an early stage of differentiation, only 9 DEGs were detected. This number increased substantially to 83 DEGs at P4, and then decreased to 12 DEGs at P7 (Fig. 1D and Tables S1-3). X- and Y-linked genes such as *Xist*, *Ddx3y*, and *Kdm5d*, were the most differentially expressed at all the stages, but the DEGs were enriched in autosomal genes. Most remarkably, at P4, only 12 of the 83 DEGs (14%) were sex-linked genes, indicating sex-biased autosomal gene expression during differentiation (Fig. 1E). Furthermore, the majority of the DEGs at this stage showed increased expression in L2/3^M^ neurons compared to L2/3^F^ neurons (Fig. 1D). Together, these results demonstrate a sexually dimorphic transcriptional regulation during L2/3 neuronal differentiation, involving both sex-linked and autosomal genes. Moreover, the data highlights P4 as the peak in sex-dependent transcriptional divergence, while the decrease in differences by P7 indicates a convergence of gene expression at later stages. 

Next, we performed over-representation analyses (ORA) to identify biological processes differentially regulated by sex at P4. This method determines whether certain biological categories (such as pathways, gene ontologies, or functional annotations) are more represented in a given list of genes than would be expected by chance. ORA of the P4 DEGs showed increased expression of genes associated with potassium ion transport, such as *Kcna2*, and with cell adhesion and extracellular matrix organization, such as *Nid1, Ndnf,* or *Slc2a10,* in L2/3^M^ neurons compared to L2/3^F^. By contrast, genes involved in fundamental processes of telencephalic development, including *Dbi*, *Neurod1,* and *Wdr62*, showed higher expression in L2/3^F^ neurons than in L2/3^M^ neurons (Fig. 1F and Tables S15,16).

In view of these findings, we compared the subsets of DEGs obtained from the prior analyses at each developmental stage to determine the genes consistently deregulated in L2/3^F^ and L2/3^M^ neurons across development. Only sex-linked genes were differentially expressed in L2/3^M^ and L2/3^F^ neurons across the three stages: five Y-linked genes (*Kdm5d*, *Eif2s3y*, *Uty*, *Ddx3y,* and *Gm20775*) and one X-linked gene (*Xist*). No autosomal genes showed consistent differences between the sexes across development. Thus, autosomal gene expression diverges in a sex dimorphic manner transiently and acutely at P4 in L2/3^M^ and L2/3^F^ neurons. Overall, our data reveal a transient peak of sex-dependent differences in gene expression in L2/3 neurons. This suggests the existence of a perinatal window of heightened susceptibility to sex-dependent neurodevelopmental phenotypes. Insults or genetic mutations that affect, directly or indirectly, the divergent transcriptional regulation of male and female L2/3 neurons during this critical period may trigger a cascade of developmental events that ultimately alter adult circuitries only in one sex.

### *Cux1* regulates L2/3 differentiation in a sex-dependent manner

**Fig. 2:**
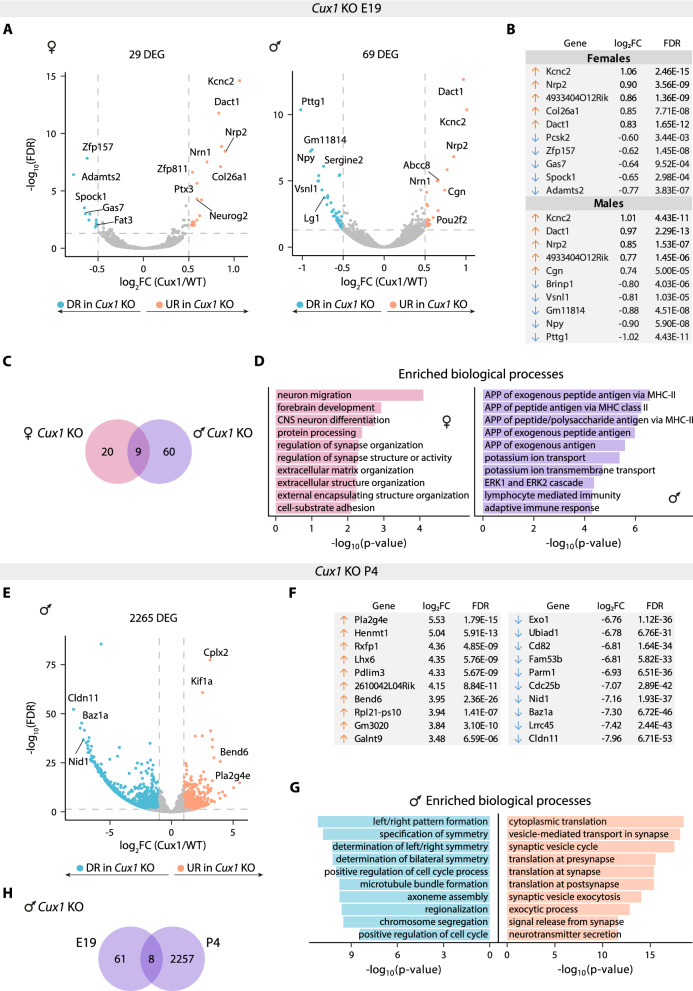
Sex- and time-dependent *Cux1* transcriptional regulation in L2/3 neurons.** A)** Volcano plots showing differentially expressed genes (DEGs) in *Cux1* KO L2/3^F^ (left) and L2/3^M^ (right) neurons compared to WT controls at E19. The effect size and direction for each gene is depicted in the x-axis as the log_2_Fold Change (log_2_FC). False Discovery Rate (FDR) values are represented on the -log_10_ scale in the y-axis. Orange and light blue dots represent up-regulated and down-regulated genes (URGs and DRGs) respectively, based on the significance threshold marked by the dashed lines (|log_2_FC|> 0.5, FDR < 0.05). **B)** Table showing the log_2_FC and FDR values of the top 5 URGs (orange arrow) and DRGs (blue arrow) in *Cux1* KO L2/3^F^ (top) and L2/3^M^ (bottom) neurons at E19. **C)** Venn diagram showing the overlap of DEGs between *Cux1* KO L2/3^F^ (magenta) and L2/3^M^ (purple) neurons at E19. **D)** Representation of up to 10 most significant gene ontology biological processes obtained from the DEGs enrichment analysis of *Cux1* KO L2/3^F^ (magenta) and L2/3^M^ (purple) neurons . APP: antigen processing and presentation. **E)** Volcano plot showing DEGs in *Cux1* KO L2/3^M^ neurons compared to controls at P4 (|log_2_FC|> 1, FDR < 0.05). **F)** Tables showing the log_2_FC and FDR values of the top 10 URGs (left) and DRGs (right) genes in *Cux1* KO L2/3^M^ neurons at P4. **G)** Representation of up to 10 most significant gene ontology biological processes obtained from URGs/DRGs of *Cux1* KO L2/3^M^ neurons at P4. Color code is maintained from the volcano plot. **H)** Venn diagram showing the overlap of DEGs between *Cux1* KO L2/3^M^ neurons at E19 (left) and P4 (right)

Our analysis revealed sex-dependent differences in the transcriptional profiles of L2/3 neurons during perinatal development. These differences peaked at P4 and diminished by P7, suggesting that male and female neurons undergo differentiation through distinct molecular trajectories that ultimately converge on a shared transcriptional state by P7. We next evaluated the effects of *Cux1* loss of function in this neuronal subpopulation. First, we compared WT and *Cux1* KO L2/3 neurons within each sex to control for sex-biased gene expression and isolate the specific effects of the KO. In E19 samples, this analysis identified 29 DEGs (19 up-regulated genes (URGs) and 10 down-regulated genes (DRGs)) in *Cux1* KO L2/3^F^ neurons and 69 DEGs (24 URGs and 45 DRGs) in *Cux1* KO L2/3^M^ neurons compared to their WT counterparts. Thus, the analysis revealed more dramatic changes in males than in females (Fig. 2A, B and Tables S4, 5). Moreover, only nine genes showed dysregulation in the two sexes. Among them, *Nrp2, Spock1*, and *Fat3* are notable for their role in neuron migration and dendritic development [11, 21, 29] (Fig. 2B, C and Table S6). As for sex-biased expression, ORA analyses showed that the most affected biological processes in *Cux1* KO L2/3^F^ neurons were related to neuronal development, whereas genes involved in ion channels and the Erk1 and Erk2 cascade were the most overrepresented in *Cux1* KO L2/3^M^ neurons (Fig. 2D and Tables S17, 18). L2/3^F^ neurons also showed a noticeable reduction of *Adamts2* expression **(**Fig. 2B and Table S4). This gene defines a subtype of L2/3 neurons in the SS cortex [14] but did not show sex-biased expression in our analysis of WT L2/3 neurons (Tables S1-3). Thus, our data indicate that CUX1 contributes to the specification of *Adamts2*^+^ L2/3 neurons only in females, while males might count on CUX1-independent regulatory cascades for an equivalent specification. The impact of *Cux1* loss of function on *Adamts2* expression is a good illustration of how differentiation trajectories can be selectively affected by an autosomal mutation only in one sex and of how these alterations might be diagnosed only upon the correct interrogation.

Next, we performed the analysis of L2/3^M^ samples at P4 and identified 2265 DEGs (836 URGs and 1429 DRGs) in *Cux1* KO vs WT (Fig. 2E, F**,** and Table S7). Comparing this data to the effects of *Cux1* KO at E19, there is a more significant impact of CUX1 deficiency over time. This may be due to a progressive opening of CUX1 binding regions during differentiation, a cascade-like amplification of the transcriptional dysregulation occurring at previous stages, or a combination of both. As we were unable to isolate L2/3^F^ samples at P4, we could not evaluate the possibility of a sex-bias component of this increased dysregulation. Interestingly, among the DRGs we found diminished expression of *Baz1a* (Fig. 2E, F**,** and Table S7)*.* This gene is expressed only in a subset of L2/3 neurons in the SS cortex and primes them for adaptive responses in sensory processing [14]. Besides *Baz1a*, *Cux1* KO also negatively impacted the expression of genes involved in microtubule formation, cell asymmetry, cytoskeleton regulation, and cilia mechanisms. Meanwhile, the analysis of URGs revealed enrichment in synaptic functions, vesicle-mediated transport, and exocytosis, all of which are in agreement with the reported functions of CUX1 in dendritic differentiation [17, 18] (Fig. 2G and Tables S19, 20).

To identify regulatory transcriptional networks commonly dysregulated across development in *Cux1* KO L2/3^M^ neurons, we compared the DEGs at E19 and P4. We identified only eight shared DEGs, which included *Npy*, *Pou2f2,* and *Fat3* (Fig. 2H and Table S6). Notably, NPY is expressed mostly, but not exclusively, in GABAergic interneurons [32], and is affected in a variety of neuropathological processes, including depression, bipolar disorder, schizophrenia, schizoaffective disorder, and Alzheimer’s disease [3, 7, 44, 45, 58, 59]. On the other hand, *Pou2f2* is known to contribute to the development of neuronal subtypes in the retina and spinal cord [31, 36], and the atypical cadherin FAT3 for controlling dendritic morphology in retinal neurons [21].

Finally, we compared the gene expression of *Cux1* KO neurons to that of WT neurons of opposite sex. This analysis evaluates the possibility that the deficiency amplifies or attenuates sex differences. Interestingly, E19 *Cux1* KO L2/3^M^ neurons were more similar to WT L2/3^F^ neurons than to WT males (27 vs 69 DEGs). This suggests that *Cux1* KO L2/3^M^ neurons shift towards a WT female-like gene expression profile (Fig. 2A and S2B, C)**.** By contrast, the same comparison at P4 showed more pronounced differences in gene expression in *Cux1* KO L2/3^M^ neurons compared to WT females than to males (2585 vs 2265 DEGs) (Fig. 2E and S2D). Notably, only 23 of these genes overlap with those exhibiting sex-biased expression in P4 WT mice (Table S12). Thus, CUX1 deficiency abolishes some of the sex-biased gene expression normally observed in male and female L2/3 neurons at E19 and induces an atypical female-biased expression at this stage that is no longer evident by P4. These findings suggest that *Cux1* may play dual and opposing roles during development—perhaps both promoting and restricting the effects of sexual hormones on L2/3 neurons. Taken together, our results demonstrate that *Cux1* KO genotype interacts with sex genotype resulting in sex-dependent phenotypes.

### Sex modifies the effects of *Cux2* knock-out during L2/3 postnatal differentiation

**Fig. 3:**
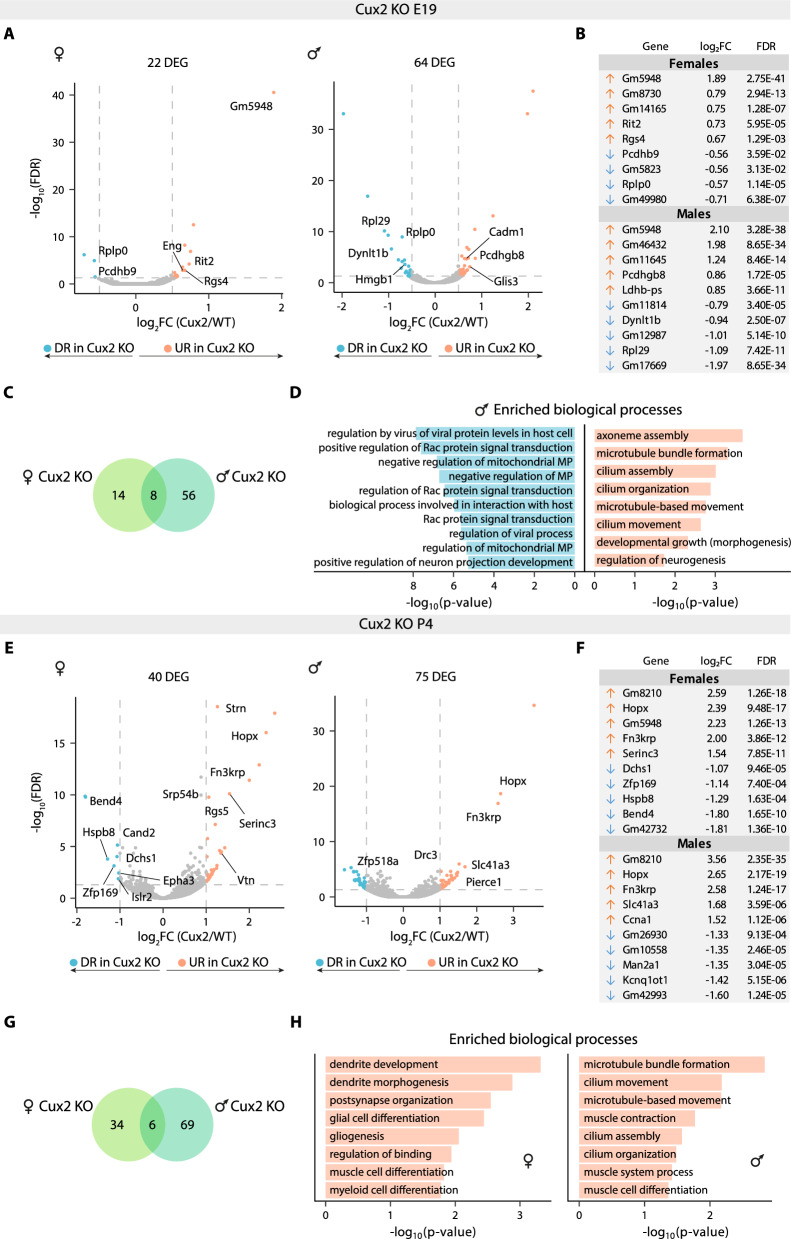
Sexual differences in *Cux2*-dependent gene expression of L2/3 neurons.** A)** Volcano plots showing differentially expressed genes (DEGs) in *Cux2* KO L2/3^F^ (left) and L2/3^M^ (right) neurons compared to controls at E19. The effect size and direction for each gene is depicted in the x-axis as the log_2_Fold Change (log_2_FC). False Discovery Rate (FDR) values are represented on the -log_10_ scale in the y-axis. Orange and light blue dots represent up-regulated and down-regulated genes (URGs and DRGs), respectively, based on the significance threshold marked by the dashed lines (|log_2_FC|> 0.5, FDR < 0.05). **B)** Table showing the log_2_FC and FDR values of the top 5 URGs (orange arrow) and DRGs (blue arrow) in *Cux2* KO L2/3^F^ (top) and L2/3^M^ (bottom) neurons at E19. **C)** Venn diagram showing the overlap of DEGs between *Cux2* KO L2/3^F^ (green) and L2/3^M^ (turquoise) neurons at E19. **D)** Representation of up to 10 most significant gene ontology biological processes obtained from the URGs (orange) and DRGs (blue) enrichment analysis of *Cux2* KO L2/3^M^ neurons at E19. MP: membrane permeability. **E)** Volcano plots showing differentially expressed genes (DEGs) in *Cux2* KO L2/3^F^ (left) and L2/3^M^ (right) neurons compared to controls at P4. Orange and light blue dots represent up-regulated and down-regulated genes (URGs and DRGs) respectively, based on the significance threshold marked by the dashed lines (|log_2_FC|> 1, FDR < 0.05). **F)** Table showing the log_2_FC and FDR values of the top 5 URGs (orange arrow) and DRGs (blue arrow) in *Cux2* KO L2/3^F^ (top) and L2/3^M^ neurons (bottom) at P4. **G)** Venn diagram showing the overlap of DEGs between *Cux2* KO L2/3^F^ (green) and L2/3^M^ (turquoise) neurons at P4. **H)** Representation of up to 10 most significant gene ontology biological processes obtained from the URGs enrichment analysis of *Cux2* KO L2/3^F^ (left) and L2/3^M^ (right) neurons at P4

We next performed a similar analysis of *Cux2* KO L2/3 neurons at E19 and P4; we could not acquire P7. The analysis of E19 samples uncovered 22 DEGs (18 URGs and four DRGs) in *Cux2* KO L2/3^F^ neurons, and 64 DEGs (40 URGs and 24 DRGs) in *Cux2* KO L2/3^M^ neurons compared to their WT counterparts (Fig. 3A, B and Tables S8, 9). Thus, as was the case for *Cux1* KO, the effect of *Cux2* KO was more dramatic in males than females at E19. Among these subsets of E19 DEGs, only eight genes were common to both sexes, including two protocadherins (*Pcdhgb5* and *Pcdhgb8*) and one ribosomal subunit (*Rplp0*) (Fig. 3C and Table S6). Interestingly, ORA did not reveal specific biological processes enriched in the DEGs of *Cux2* KO L2/3^F^ neurons, indicating overall subtle effects of CUX2 deficiency in females at this stage. In contrast, in *Cux2* KO L2/3^M^ neurons, the downregulation of several *Dynlt1* isoforms, together with the up-regulation of *Kndc1,* a gene associated with dendrite arborization [34], support the previously reported role of *Cux2* in dendritogenesis [17]. Conversely, microtubule-related processes were upregulated, perhaps as a compensatory mechanism (Fig. 3D and Tables S9, 21, 22).

At P4, we found 40 DEGs (32 URGs and 8 DRGs) in *Cux2* KO L2/3^F^ neurons and 75 DEGs (42 URGs and 33 DRGs) in *Cux2* KO L2/3^M^ neurons compared to their WT counterparts (Fig. 3E, F**,** and Tables S10, 11). Hence, although the overall dysregulation of gene expression is lesser in CUX2 than CUX1 deficiency, in both conditions the differences increase with differentiation and are more pronounced in males than in females. Moreover, only six genes were commonly dysregulated in both sexes in *Cux2* KO neurons, indicating an interaction of the *Cux2* KO genotype with sex genotype (Fig. 3G and Table S6). Regarding dysregulated functions, URGs in *Cux2* KO L2/3^F^ neurons were enriched in genes regulating dendrite development and organisation of postsynaptic structures (e.g., *Sult4a1* or *Xlr3b*), while those in *Cux2* KO L2/3^M^ neurons were enriched in cilium and microtubule-associated processes (Fig. 3H and Tables S10, 23,24).

To further evaluate the interference of CUX2 deficiency with sex-dependent differentiation, we compared gene expression in *Cux2* KO L2/3^F^ and L2/3^M^ neurons. This showed differences of only eight and seven genes at E19 and P4, respectively. Most of these DEGs were sex-linked genes—only *Foxj1* and *1700003E16Rik* were autosomal (Fig. S2E, H). These data indicate that although the dysregulation of gene expression is more pronounced in males, females follow a similar directional shift in gene expression.

When comparing to WT, E19 *Cux2* KO L2/3^M^ neurons showed a more similar transcriptome to WT L2/3^F^ neurons than to WT L2/3^M^ neurons (42 vs 64 DEGs). Meanwhile, gene expression in E19 *Cux2* KO L2/3^F^ neurons departed more from WT L2/3^M^ neurons than from WT L2/3^F^ neurons (31 vs 22 DEGs) (Fig. S2F, G). These data again support a continuum of dysregulated gene expression profiles. At P4, the transcriptomes of both *Cux2* KO L2/3^F^ and L2/3^M^ neurons were closer to those of WT L2/3 neurons of opposite sex than to those of the same sex, reflecting incomplete or intermediate sex-biased gene expression (Fig. S2I, J). In sum, these analyses indicate that loss of *Cux2* cancels aspects of sex-dependent L2/3 differentiation and equalizes the process to form intermediate in-between male–female transcriptome stages. Hence, the data suggest a role of *Cux2* in ensuring sex-dependent differentiation in cortical neurons. In this regard, it should be noted that in this study we used a *Cux2* germ-line KO, and thus, the phenotypes could be also non-cell autonomous.

### *Cux1* and *Cux2* regulate L2/3 neuronal development through non-redundant networks

**Fig. 4:**
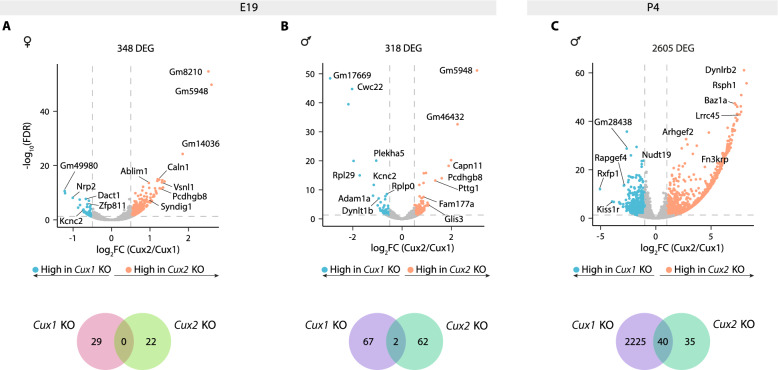
Non-redundant regulatory networks of *Cux* genes during L2/3 neuronal development. **A-C**) (Top) Volcano plots showing differentially expressed genes (DEGs) in *Cux2* KO neurons compared to *Cux1* KO at E19 (L2/3^F^ (**A**) and L2/3^M^ (**B**) and between *Cux2* KO and *Cux1* KO L2/3^M^ neurons at P4 (**C**). The effect size and direction for each gene is depicted in the x-axis as the log_2_Fold Change (log_2_FC). False Discovery Rate (FDR) values are represented on the -log_10_ scale in the y-axis. Orange and light blue dots represent up-regulated and down-regulated genes (URGs and DRGs) respectively, based on the significant threshold marked by the dashed lines (|log_2_FC|> 0.5 for E19 and |log_2_FC|> 1 for P4, FDR < 0.05). (Bottom) Venn diagrams showing the overlap of DEGs between *Cux1* KO and *Cux2* KO neurons at E19 (L2/3^F^ (**A**) and L2/3^M^ (**B**) and at P4 (**C**)

Paralogous genes, such as *Cux1* and *Cux2*, arise by gene duplication during evolution to buffer and innovate parental gene functions. To evaluate redundant and non-redundant transcriptional activities of the CUX proteins, we assessed the similarities and differences in the transcriptional fingerprints of *Cux1* and *Cux2* KOs. The comparison of E19 *Cux2* KO and E19 *Cux1* KO transcriptomes identified 348 DEGs when comparing L2/3^F^ neurons and 318 DEGs in L2/3^M^ neurons (Fig. 4A, B). Thus, differences are observed in both sexes, with non-redundant transcriptional functions of *Cux1* and *Cux2*, in agreement with previous analyses of cellular and functional phenotypes [16–18, 76]. Moreover, there was no significant overlapping of the DEGs of *Cux1* vs WT and *Cux2* KO vs WT in females and males (Fig. 4A, B). Only in L2/3^M^ neurons did we identify two genes that were consistently dysregulated: *Gm11814* (a pseudogene), and *Slc44a5*a (a solute carrier membrane protein) (Table S6). Although we could not compare female samples at P4, the comparison of the male samples revealed that the transcriptional profiles of *Cux2* KO and *Cux1* KO L2/3 neurons diverge further at P4, with 2605 DEGs (Fig. 4C and Table S6). Together, these analyses add to previous evidence that *Cux1* and *Cux2* regulate the development of L2/3 neurons through broadly non-redundant targets or genetic cascades.

### Segregated sex analyses are required to identify sex differences in early development

**Fig. 5:**
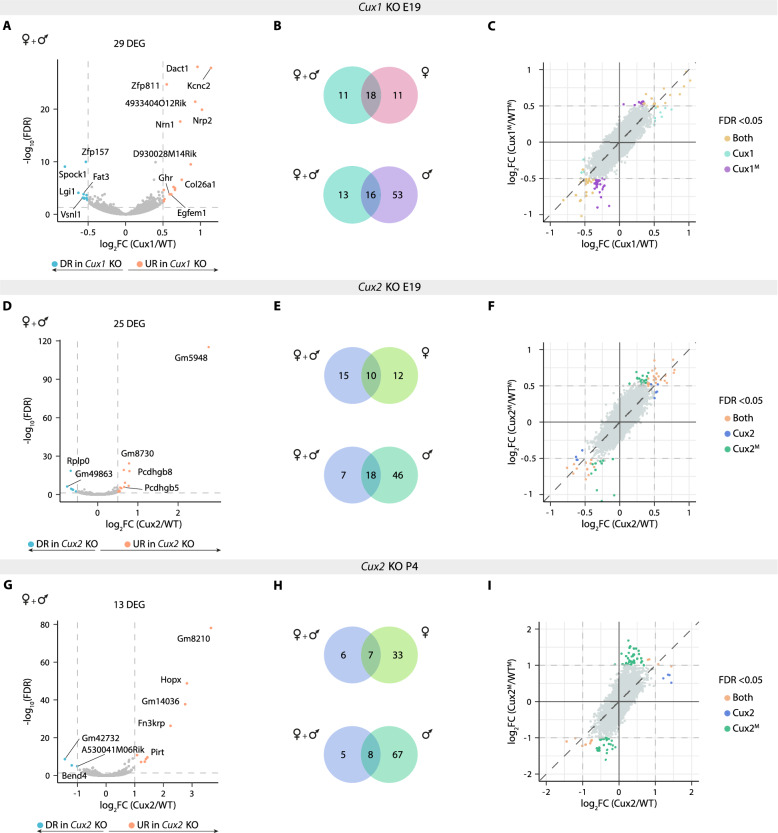
Differences in gene expression profiles in *Cux* KO neurons between mixed-sex and sex-dependent analyses. **A, D, G)** Volcano plots showing differentially expressed genes (DEGs) in a mixture of *Cux* KO L2/3^F^ and L2/3^M^ neurons (mixed-sex analysis): *Cux1* KO at E19 (A), and *Cux2* KO at E19 (D) and at P4 (G) compared to WTs. The effect size and direction for each gene is depicted in the x-axis as the log_2_Fold Change (log_2_FC). False Discovery Rate (FDR) values are represented on the -log_10_ scale in the y-axis. Orange and light blue dots represent up-regulated and down-regulated genes (URGs and DRGs) respectively, based on the significant threshold. Dashed lines mark the threshold in each plot: A, D, |log_2_FC|> 0.5, FDR < 0.05; G, |log_2_FC|> 1, FDR < 0.05. **B, E, H)** Venn diagrams showing the overlap of DEGs between mixed-sex and sex-dependent gene expression analyses in E19 *Cux1* KO (B), E19 *Cux2* KO (E), and P4 *Cux2* KO L2/3 neurons (H). **C, F, I)** Scatter plots of log_2_FC values obtained in the mixed-sex analysis and the one exclusively conducted on male samples for E19 *Cux1* KO (C), E19 *Cux2* KO (F), and P4 *Cux2* KO (I). Color code indicates statistical significance (FDR < 0.05) in one or both analyses. Grey dots represent non-significant genes in either mixed-sex or sex-dependent analyses: C, F,|log_2_FC|< 0.5, FDR > 0.05; G, |log_2_FC|< 1, FDR > 0.05

Our final comparison assessed the extent to which pooling samples across sexes may obscure sex-dependent transcriptional signatures. For this purpose, we reanalyzed the data after pooling samples regardless of sex, although for *Cux1* KO L2/3 neurons it was limited to E19 datasets due to the absence of female samples at P4. This approach identified many of the DEGs found in the sex-specific analyses and even revealed some additional genes, likely due to increased statistical power. However, it also masked many DEGs that were evident when males and females were analyzed separately (Fig. 5A, B, D, E, G, H, and Tables S12-14).

To better evaluate and illustrate the advantages of each type of analysis, we plotted the fold change values (log₂FC) for each gene obtained from the mixed-sex analysis against those from the male-only analysis (Fig. 5C, F, and I). Most genes, whether dysregulated (|log₂FC ≥ 0.5|) or not, clustered near the diagonal, indicating that similar values of expression differences were obtained regardless of the samples included in the analysis. However, many genes with a significant differential expression profile in the male analysis deviated from the diagonal, reflecting the substantial difference in the results obtained in one-sex or mixed-sex analyses. This masking effect was particularly pronounced in the male-only datasets and more frequent for DRGs than for URGs (Fig. 5C, F, and I). In contrast, most genes detected only in the mixed-sex analysis had similar values in the male analysis, but statistical significance was only achieved when all samples were included. Hence, in a mixed analysis the increased resolution is due to sample size rather than a biological effect. On the other hand, pairing the samples according to sex may decrease statistical power but allows identification of sex-biased dysregulation. Taken together, our comparisons demonstrate that, across all KOs and developmental stages, mixed-sex analyses tend to obscure genes that are highly dysregulated in *Cux* KO L2/3^M^ neurons.

## Discussion

The role of sexual dimorphism in the development of neuronal circuits remains poorly explored. It is broadly acknowledged that for NDDs, the underlying cause may originate long before the onset of clinical symptoms, and growing evidence points to the importance of identifying the developmental timing of the defects caused by specific gene mutations [88]. Here, we characterized the dynamic changes in the transcriptional expression of L2/3 cortical neurons across three key developmental stages—E19, P4, and P7—and identified a transient window of sex-biased gene expression at P4. Furthermore, we found a sex-biased dysregulation of gene expression across this developmental window in *Cux1* and *Cux2* KO L2/3 neurons. For clinical studies, with the predictable expansion of patient cohorts, our results pave the way for the future investigation of sex differences in *CUX* linked NDDs, including ASD, intellectual disability, and epilepsy [1, 2, 12, 33, 48, 67, 100].

Building on the example of *CUX* linked NDDs, our findings underscore both the importance of incorporating sex genotype and evaluating developmental trajectories in the analysis of neurodevelopmental disorders. We propose that P4 represents a window of sex-dependent developmental vulnerability, during which genetic perturbations may exert their effects. The analysis of the transcriptional changes triggered by the disruption of two key TFs, CUX1 and CUX2, show a heightened susceptibility of male neurons to genetic disruptions affecting *Cux1/2*-dependent pathways during this developmental window, supporting our hypothesis.

Our results align with growing evidence of sex-biased gene expression in the human brain [46, 92], which may emerge only within specific neuronal populations and/or developmental windows. In fact, we show that sex differences in gene expression are best analyzed across development and can only be uncovered when sex genotype is explicitly considered as a variable. Furthermore, the disadvantages of segregating the analysis by sex can be easily counteracted by increasing the number of samples or by including parallel mixed-sex analysis. Thus, as many NDDs exhibit cell type-specific vulnerabilities [37, 79], sex is a strong variable to consider when studying these disorders.

The conditional approach used to generate *Cux1* KO L2/3 neurons demonstrates that sex-biased transcriptional dysregulation can originate intrinsically in an otherwise WT animal context. Only the isolated L2/3 neurons lack expression of CUX1 protein, and thus the phenotypes that emerge do so independently of defects in other cell types or tissues, such as the gonads, liver, or any other organ showing sexual dimorphisms that we could encounter in a germ-line KO. Hence, cell-autonomous interactions of Y- or X-linked genes with *Cux* mutations could contribute to explain sex biases. Along this line, a recent study found sex-biased gene expression in human embryonic stem cells as well as in immature neurons [70]. Similar to our results in CUX-deficient neurons, male cells showed more divergent transcriptomes, with DEGs being both Y-linked and autosomal genes. Notably, this study highlighted the key role of two Y-linked demethylases, UTY and KDM5D, in these differences [70], which we also found as sex-biased DEGs during the early postnatal differentiation of WT L2/3 neurons. Therefore, these chromatin remodelers may contribute to the observed phenotypes.

It should be noted that the sex-dependent DEGs identified in *Cux* mutants are not confined to genes exhibiting sex-biased expression in WT mice. Instead, the sex-biased effects of *Cux* mutations appear to arise from a broader perturbation of molecular cascades that are ordinarily sex-independent. For example, *Adamts2,* and *Npy* emerge as downstream targets of *Cux1* at P4 specifically in females and males, respectively, despite neither gene exhibiting sex-biased expression in WT conditions at any developmental stage. Thus, their dysregulation in *Cux1* KO neurons reflects a shift from unbiased to sex-biased gene expression, a phenomenon also observed in *Cux2* mutants. Future research is needed to characterize this sex-specific dysregulation of sex-independent gene cascades.

While our analysis clearly demonstrates the importance of considering cell-type specific mechanisms underlying NDD symptoms, we do not exclude the contribution of non-cell-autonomous interactions into the observed phenotypes. Early in development, sex hormones trigger distinct organizational and signaling changes in the brain. In males, these changes lead to a male-specific neurochemical profile and synaptic connectivity that result in the masculinization and defeminization of the brain, eventually promoting male-like behaviors while limiting female-typical behavioral potential [54, 83]. In male mice, serum testosterone levels rise at E16 and decline shortly after birth, and this early hormonal surge is considered one of the primary factors driving the masculinization of the brain [15, 60] The marked sex-biased expression of autosomal genes observed at P4 may reflect masculinization processes, perhaps representing long-term cascading effects of the postnatal testosterone surge rather than its direct effects. While a detailed mechanistic analysis was beyond the scope of the present study, sex-biased gene expression could also arise from other influences, such as the loss of maternal estrogen during the first postnatal week or more directly intrinsic genetic programs. Most notably, the transient nature of the changes observed in WT neurons highlights the complex regulatory mechanisms at play in sex-specific brain differentiation.

Despite its strengths, this study has some limitations that affect the interpretations of the results. First, the temporal coverage of the mutant analyses is incomplete due to the increased fragility of *Cux1* and *Cux2* KO neurons during the dissociation protocol. This limitation reduces our ability to fully resolve the temporal trajectory of the transcriptional dysregulation of L2/3 neurons in these mutants. Second, because *Cux2* is expressed in urogenital tissue, we cannot rule out a hormonal imbalance as a possible explanation of the phenotypes in L2/3 neurons from *Cux2* KO mice [35].

The perinatal window of neonatal development is increasingly recognized as a critical diagnostic and therapeutic window for disorders of sex development (DSDs) and possibly for NDDs [10, 51, 77, 84]. Our study supports the latter and suggests that transcriptional profiling during this window may reveal early biomarkers of sex-dependent vulnerability, particularly in a cell type- and layer-specific context. Our findings provide compelling evidence for a sex-specific window of transcriptional plasticity in L2/3 neurons and suggest that this period may be particularly sensitive to genetic perturbations. The results highlight the need for transcriptomic analysis of specific neuronal types segregated by sex. Such approaches appear crucial for the identification of sex-specific mechanisms of disease, windows of vulnerability, and targeted therapeutic strategies that would be invisible in less granular analyses.

## Supplementary Information

Additional file 1: Figure S1. Validation of *Cux1* conditional knock-out mouse model. **A)** DNA constructions employed in generating and characterizing *Cux1*^*f*^ and *Cux1*^*Δ*^^*23*^ alleles. **B)** Schematic representation of *Cux1* used in this study. A ssDNA containing *Cux1* exon 23 sequence flanked by two loxP sequences was used to generate *Cux1* conditional allele (*Cux1*^*f*^) by Easi-CRISPR. *Cux1* KO allele (*Cux1*^*Δ23*^) is generated due to Cre-mediated recombination of the loxP sequences in *Cux1*^*f*^ allele. **C)** Confocal images showing *Cux1* subcellular distribution in WT and *Cux1*^f/^^Δ23^ mice. Mice were subjected to IUE at E15.5 with pCAG-Cre and pCALNL-GFP and brains were analyzed at P21. White and blue arrows highlight electroporated and non-electroporated neurons respectively. (Green = GFP, Magenta = *Cux1*, Blue = DAPI. Scale bar = 10 um). **D)** Western blot showing cortical expression of *CUX1* p200 in *Cux1*^*+/f*^, *Cux1*^*+/*^^*Δ23*^,*Cux1*^*f/*^^*Δ23*^ and *Cux1*^*Δ23/Δ23*^ mice at E18.5. α-tubulin was used as loading control. Images in panels (C,D) are representative of two biological replicates. Figure S2. Opposite sexual approach of *Cux* KO transcriptional profiles in L2/3 neurons. **A-C)** Volcano plots showing differentially expressed genes (DEGs) in *Cux1* KO L2/3 neurons between sexes (A) and compared to the opposite controls (B, C) at E19. **D)** Volcano plot showing DEGs in *Cux1* KO L2/3^M^ neurons compared to L2/3^F^ controls at P4. **E-G)** Volcano plots showing DEGs in *Cux2* KO L2/3 neurons between sexes (E) and compared to the opposite controls (F, G) at E19. **H-J)** Volcano plots showing DEGs in *Cux2* KO L2/3 neurons between sexes (H) and compared to the opposite controls (I, J) at P4. The effect size and direction for each gene is depicted in the x-axis as the log_2_Fold Change (log_2_FC). False Discovery Rate (FDR) values are represented on the -log_10_ scale in the y-axis. Orange and light blue dots represent up-regulated and down-regulated genes (URGs and DRGs) respectively, based on the significant threshold marked by the dashed lines (A-C, E-G, |log_2_FC|>0.5, FDR<0.05; D, H-J, |log_2_FC|>1, FDR<0.05). Green and purple names represent Y- and X-linked genes respectively. Uncropped Gels and Blots images.

Additional file 2. Supplementary Tables S1-14. Related to Figures 1 to 5. List and information of differentially expressed genes compared as indicated in the upper cell on each individual table-sheet.

Additional file 3. Supplementary Tables S15-21. Related to Figures 1 to 5. Overrepresentation analysis of differentially expressed genes in the different comparisons as indicated in the upper cell on each individual table-sheet.

## Data Availability

The data discussed in this publication have been deposited in NCBI’s Gene Expression Omnibus [25] and are accessible through GEO Series accession number [GSE310138](http://www.ncbi.nlm.nih.gov/geo/query/acc.cgi?acc=GSE310138). All scripts used for data processing and analysis are available in the following GitHub repository: https://github.com/emarcosgra/RNAseq-scripts
