# Supplementary material for: A transient sex-biased transcriptional program shapes early postnatal L2/3 neuron development
Source: Biol Sex Differ. 2026 Apr 8;17:107. doi: 10.1186/s13293-026-00885-x (PMC13173949; doi:10.1186/s13293-026-00885-x)
Supplement: Supplementary file 1 — Additional file 1: Figure S1. Validation of Cux1 conditional knock-out mouse model. A) DNA constructions employed in generating and characterizing Cux1f and Cux1Δ23 alleles. B) Schematic representation of Cux1 used in this study. A ssDNA containing Cux1 exon 23 sequence flanked by two loxP sequences was used to generate Cux1 conditional allele (Cux1f) by Easi-CRISPR. Cux1 KO allele (Cux1Δ23) is generated due to Cre-mediated recombination of the loxP sequences in Cux1f allele. C) Confocal images showing Cux1 subcellular distribution in WT and Cux1f/Δ23 mice. Mice were subjected to IUE at E15.5 with pCAG-Cre and pCALNL-GFP and brains were analyzed at P21. White and blue arrows highlight electroporated and non-electroporated neurons respectively. (Green = GFP, Magenta = Cux1, Blue = DAPI. Scale bar = 10 um). D) Western blot showing cortical expression of CUX1 p200 in Cux1+/f, Cux1+/Δ23,Cux1f/Δ23 and Cux1Δ23/Δ23 mice at E18.5. α-tubulin was used as loading control. Images in panels (C,D) are representative of two biological replicates. Figure S2. Opposite sexual approach of Cux KO transcriptional profiles in L2/3 neurons. A-C) Volcano plots showing differentially expressed genes (DEGs) in Cux1 KO L2/3 neurons between sexes (A) and compared to the opposite controls (B, C) at E19. D) Volcano plot showing DEGs in Cux1 KO L2/3M neurons compared to L2/3F controls at P4. E-G) Volcano plots showing DEGs in Cux2 KO L2/3 neurons between sexes (E) and compared to the opposite controls (F, G) at E19. H-J) Volcano plots showing DEGs in Cux2 KO L2/3 neurons between sexes (H) and compared to the opposite controls (I, J) at P4. The effect size and direction for each gene is depicted in the x-axis as the log2Fold Change (log2FC). False Discovery Rate (FDR) values are represented on the -log10 scale in the y-axis. Orange and light blue dots represent up-regulated and down-regulated genes (URGs and DRGs) respectively, based on the significant threshold marked by the dashed lines (A-C, E- [file 13293_2026_885_MOESM1_ESM.pdf]

Supplementary Information

Title: A Transient Sex-Biased Transcriptional Program Shapes Early Postnatal L2/3 Neuron Development

Authors: Elia Marcos-Grañeda[1], Fernando Martín-Fernandez[1], Linnea A. Weiss[1,2], Juan C. Oliveros[3], and Marta Nieto[1*]

Affiliations: [1]Department of Molecular and Cellular Biology, Centro Nacional de Biotecnología, Consejo Superior de Investigaciones Científicas (CNB-CSIC), Madrid, 28049, Spain. [2]Present address: Department of Genetics, Yale University, Yale School of Medicine, New Haven, CT 06510, United States. [3]Bioinformatics for Genomics and Proteomics Unit, Consejo Superior de Investigaciones Científicas (CNB-CSIC), Madrid, 28049, Spain.

*Correspondence: mnlopez@cnb.csic.es

Supplementary Materials and Methods

Generation of *Cux1* conditional knock-out mice

Cux1 conditional knock-out allele $Cux1^{em1cnbbm}$, hereafter $Cux1^{f}$, was developed following *Easi*-CRISPR strategy (98,99). Briefly, Breaking-Cas tool (100) was employed to design crRNA sequences targeting introns 22 and 23, using as reference a consensus coding sequence of *Cux1* (ENSMUST00000004097, CCDS39328) obtained from Ensembl (101). 5´-crRNA 5´-CATATAGTCTGAGTGTGGTA-3´ and 3´-crRNA 5´-GTCCATCCCAAGGTCCTATA-3´ were in silico synthesized (IDT). 2:1 proportion of crRNAs and tracrRNA (IDT, #1072533) were incubated in 5 min at 95ºC to generate guide RNA (gRNA) complexes. ssDNA was also designed using the *Cux1* consensus coding sequence. It was synthesized by Megamer ssDNA Fragments (IDT) using a 950 bp ssDNA that contained two loxP sequences located 137 bp 5´ and 201 bp 3´of exon 23 and two 110 bp homology arms. EcoRV and XbaI single restriction sites were also inserted near 5´ and 3´ loxP sequences respectively. Mouse transgenesis was performed in the Transgenesis Unit of the CNB. 8-cell embryos were microinjected with a mix of gRNA:Cas9 complexes and ssDNA and transferred to pseudopregnant B6/CBA mice. The offspring was selected by genotyping the $Cux1^{f}$ conditional allele using forward 5´-CTCTGCCAGCAGGGTATTCAC-3´ and reverse 5´-GTAGGCTTTGGAGGGTGTAGG-3´ primers. Repeated crosses with $C57BL6J^{RccHsd}$ mice were set to obtain a congenic mouse strain (B6.Cg-$Cux1^{f}$). Mice were also crossed with hemizygous Sox2-Cre mice (B6.Cg-$^{Edil3Tg(Sox2-Cre)1Amc/}$J, JAX stock #008454) to generate heterozygous knock-out mice ($Cux1^{f/\Delta 23}$).

Immunofluorescence

Mice were anesthetized using an intraperitoneal injection of ketamine/xylazine and perfused with formalin (Merck, #HT501128). Brains were postfixed in formalin, cryoprotected in 30% sucrose, and cut in 50 µm free-floating cryosections. Primary antibodies used were mouse anti-GFP (Roche, #11814460001) and rabbit anti-Cux1 (Santa Cruz Biotechnology, #sc-13024X); and secondary antibodies were goat anti-rabbit Alexa 488 (Thermo Fisher Scientific, #A11034) and goat anti-mouse 647 (Thermo Fisher Scientific, #A21236). Antibodies were used at 1:500 dilution in PBS-Tween. Nuclei were stained with 4’,6-diamidino-2-phenylindole (DAPI) (Merck, #D9542).

Confocal imaging

Confocal microscopy was performed with a TCS-SP5 (Leica). Sections (145 µm) were obtained by taking 0.5 µm serial optical sections with LAS AF v1.8 software (Leica) using a 1024 x 1024 scan format with a 100x objective. The maximum threshold was set by conditions reaching signal saturation in WT.

Western blot

Cortical shells from E18 animals were isolated in cold PBS, anterior and posterior thirds were dissected away, and the remaining third, roughly corresponding to SS cortex, was snap-frozen. Tissue was lysed using four freeze-thaw cycles and homogenization with a 25 gauge needle in buffer containing 50 mM Tris-HCl pH7.5, 1% Triton X-100, 1 mM EDTA, 1 mM EGTA, 50 mM NaF, 10 mM β-glycerophosphate, 5 mM $Na_4P_2O_7$, 1 mM Na_3VO_4, 0.27 M sucrose, 1 mM PMSF, and protease inhibitor cocktail. 20 µg of total protein were separated under denaturing condition in laemmli buffer in a 4-15% gradient polyacrylamide-SDS gel (Bio-Rad, #4561083) and transferred to PVDF membranes (Merck, #IPVH00010). Membranes were blocked for one hour in tris-buffered saline with 5% milk TBS-T, and incubated overnight with primary antibodies diluted in 3% milk TBS-T (1:1000 anti-Cux1, Proteintech #11733-1-AP; or 1:2000 anti-α-tubulin, Sigma #T5168). Next, they were washed and incubated in secondary HRP-conjugated antibodies. Chemiluminescent detection was performed using SuperSignal West Pico PLUS (ThermoFishe, #34577) and a ChemiDoc Imaging System (Bio-Rad).

Supplementary table legends

Supplementary tables S1-S14. Related to Figure 1 to 5. List and information of Differentially expressed genes compared as indicated in the upper cell on each individual table-sheet.

Supplementary tables S15-S24. Related to Figure 1 to 5. Overrepresentation analysis of up-regulated genes in the different comparisons as indicated in the upper cell on each individual table-sheet.

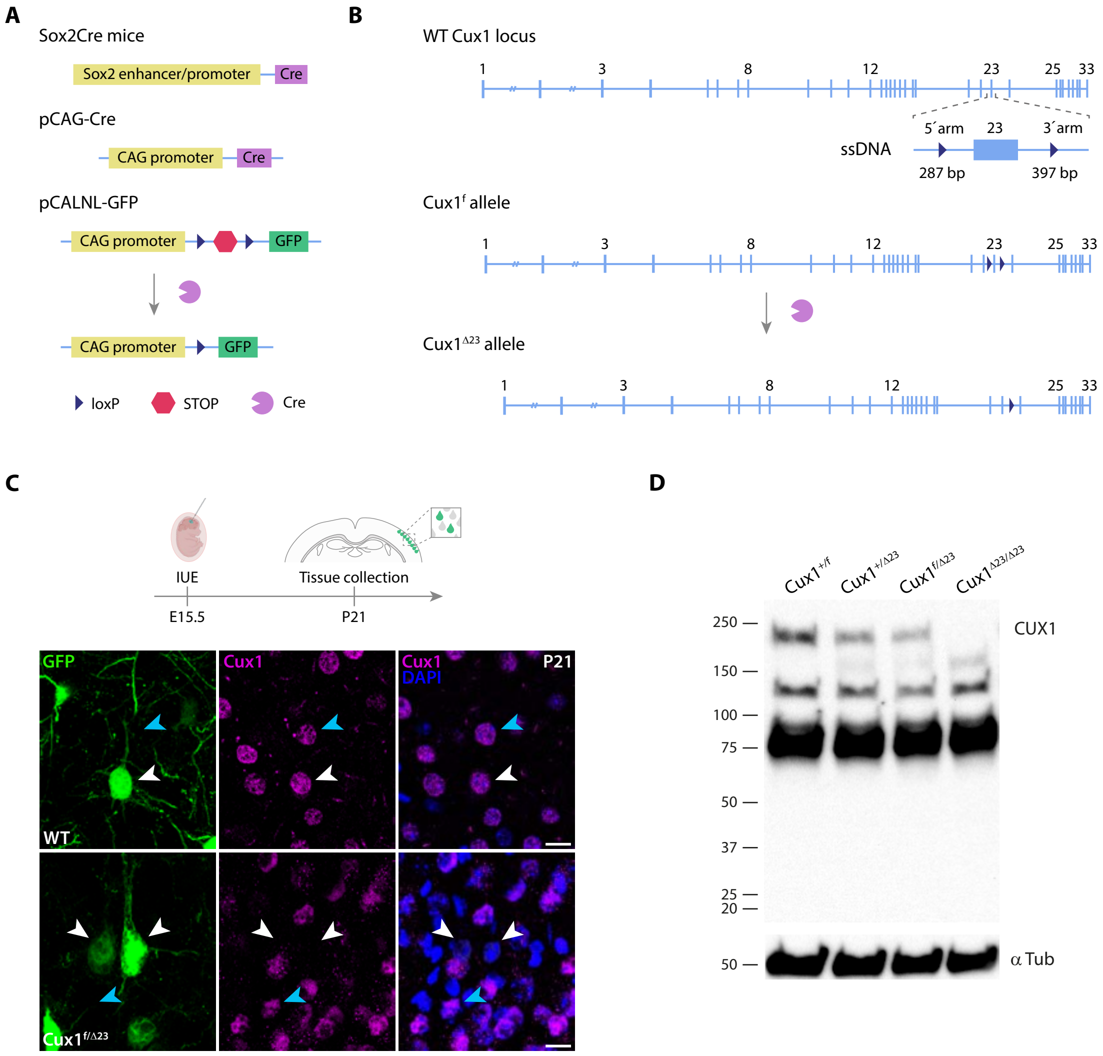

Figure S1. Validation of Cux1 conditional knock-out mouse model. **A)** DNA constructions employed in generating and characterizing Cux1^{f} and Cux1$^{\Delta23}$ alleles. **B)** Schematic representation of Cux1 used in this study. A ssDNA containing Cux1 exon 23 sequence flanked by two loxP sequences was used to generate Cux1 conditional allele (Cux1^{f}) by Easi-CRISPR. Cux1 knock-out allele (Cux1$^{\Delta23}$) is generated due to Cre-mediated recombination of the loxP sequences in Cux1^{f} allele. **C)** Confocal images showing Cux1 subcellular distribution in WT and Cux1$^{f/\Delta23}$ mice. Mice were subjected to IUE at E15.5 with pCAG-Cre and pCALNL-GFP and brains were analysed at P21. White and blue arrows highlight electroporated and non-electroporated neurons respectively. (Green=GFP, Magenta=Cux1, Blue=DAPI. Scale bar = 10 μm). **D)** Western blot showing cortical expression of CUX1 p200 in Cux1$^{+/f}$, Cux1$^{+/\Delta23}$, Cux1$^{f/\Delta23}$ and Cux1$^{\Delta23/\Delta23}$ mice at E18.5. α-tubulin was used as loading control. Images in panels (C,D) are representative of two ibiological replicates.

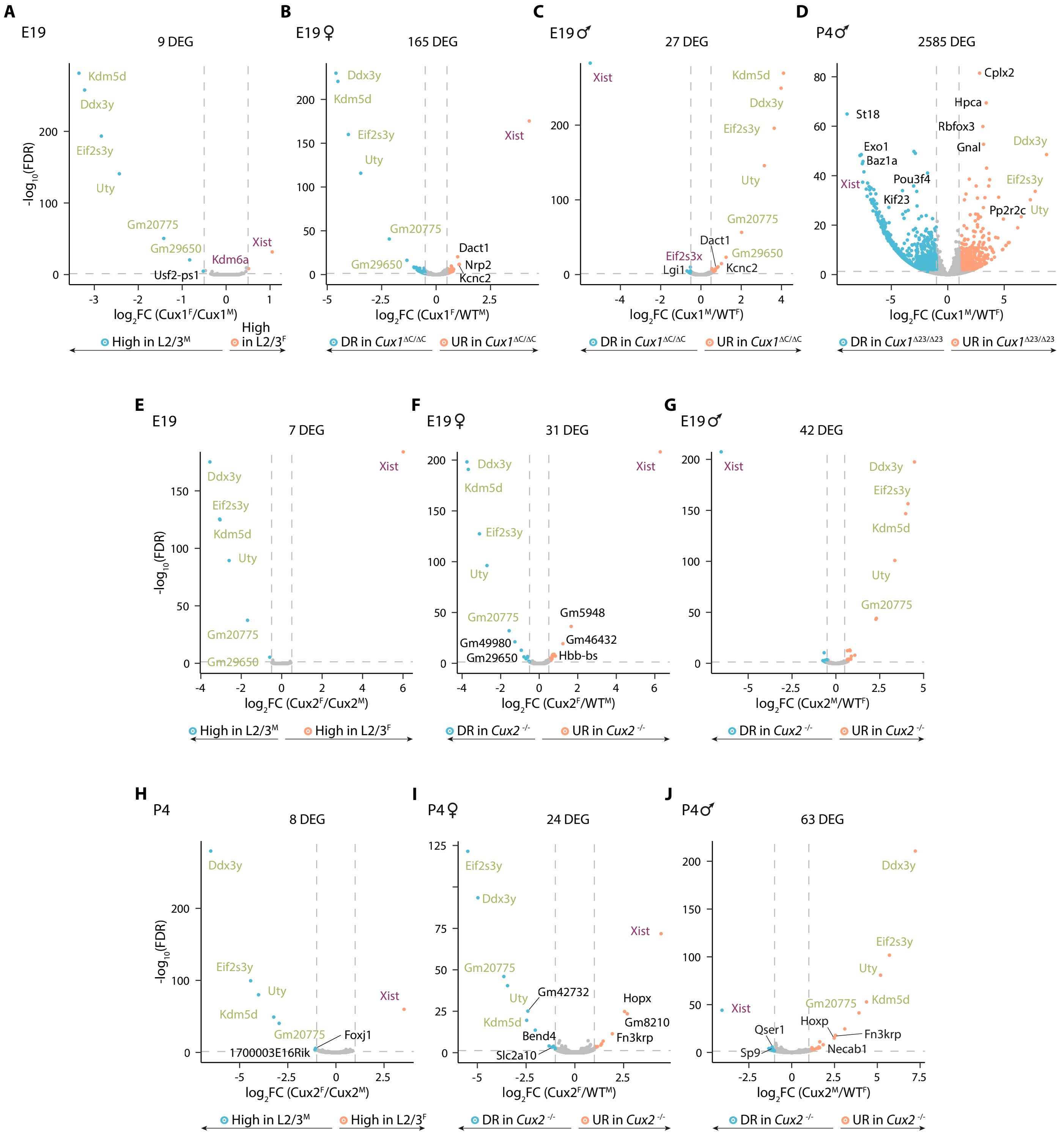

Figure S2. Opposite sexual approach of Cux knock-out transcriptional profiles in SSL2/3 neurons. A-C) Volcano plots showing differentially expressed genes (DEGs) in Cux1$^{\Delta C/\Delta C}$ L2/3 neurons between sex (**A**) and compared to the opposite controls (**B**,**C**) at E19. **D)** Volcano plot showing DEGs in Cux1$^{\Delta 23/\Delta 23}$ L2/3^{M} neurons compared to L2/3^{F} controls at P4. **E-G)** Volcano plots showing DEGs in Cux2$^{-/-}$ L2/3 neurons between sex (**E**) and compared to the opposite controls (**F**,**G**) at E19. **H-J)** Volcano plots showing DEGs in Cux2$^{-/-}$ L2/3 neurons between sex (**H**) and compared to the opposite controls (**I**,**J**) at P4. The effect size and direction for each gene is depicted in the x-axis as the $\log_2$Fold Change ($\log_2$FC). False Discovery Rate (FDR) values are represented on the -$\log_{10}$ scale in the y-axis. Orange and light blue dots represent up-regulated and down-regulated genes (URGs and DRGs) respectively, based on the significant threshold marked by the dashed lines (A-C, E-G, $|\log_2FC|>0.5$, FDR<0.05; D, H-J, $|\log_2FC|>1$, FDR<0.05). Green and purple names represent Y- and X-linked genes respectively.

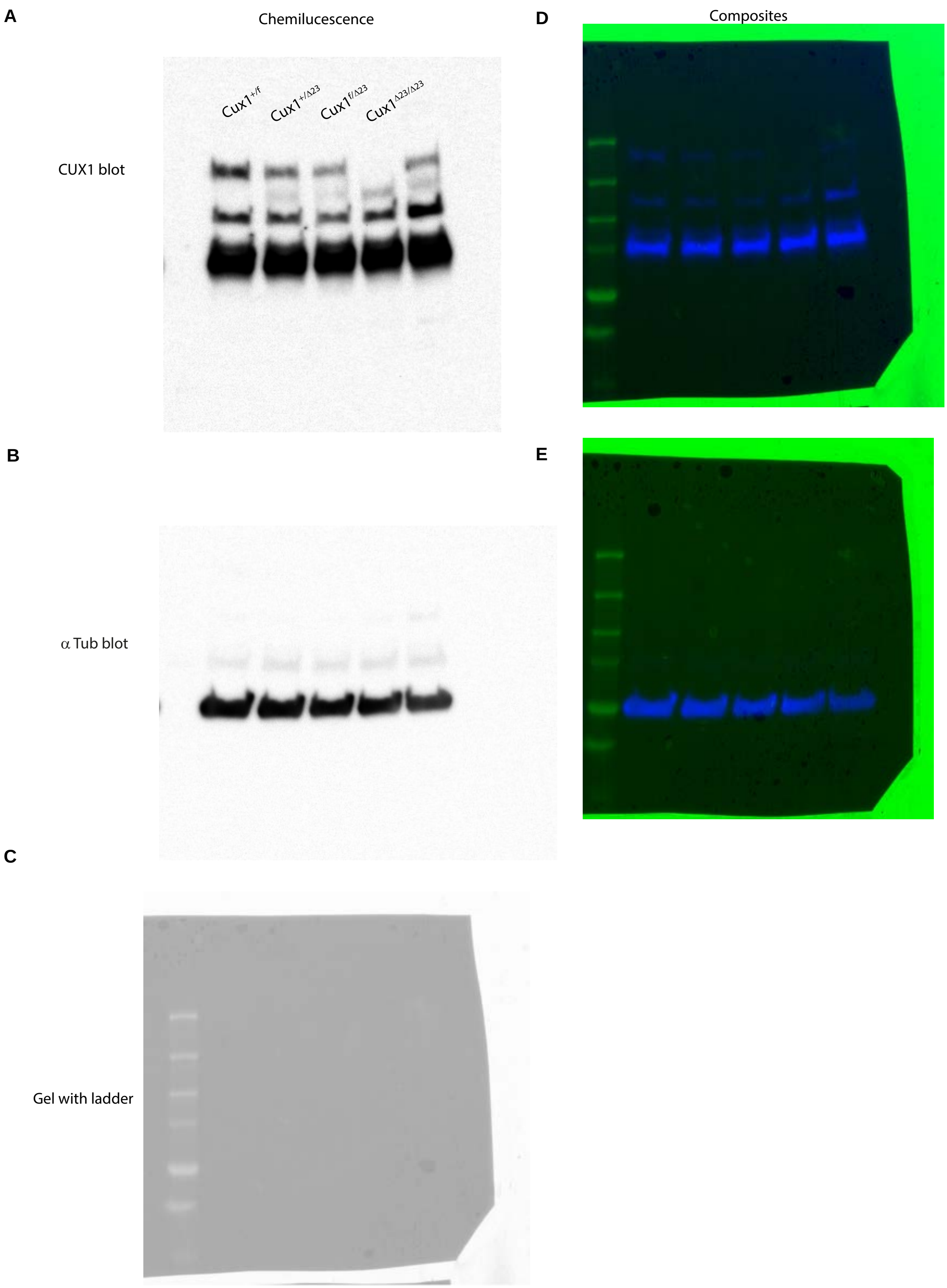

Figure S3. Original uncropped Western blot membranes for the experiments shown in Figure S1. Unprocessed blots with anti-Cux1 (A), anti-alpha-tubulin (B), gel with ladder(C), and composite superimpositions of the corresponding blots and gel with ladder (D and E).
